# Risk of overhydration and low lean tissue index as measured using a body composition monitor in patients on hemodialysis: a systemic review and meta-analysis

**DOI:** 10.1080/0886022X.2017.1419963

**Published:** 2018-01-19

**Authors:** Seun Deuk Hwang, Jin Ho Lee, Seoung Woo Lee, Joong Kyung Kim, Moon-Jae Kim, Joon Ho Song

**Affiliations:** a Division of Nephrology and Hypertension, Department of Internal Medicine, Inha University College of Medicine, Incheon, Korea;; b Divison of Nephrology, Department of Internal Medicine, Bong Seng Memorial Hospital, Busan, Korea

**Keywords:** Overhydration, lean tissue index, body composition monitor, hemodialysis, end stage renal disease

## Abstract

Overhydration and sarcopenia, related to an individual’s nutritional status, have been associated with increased cardiovascular mortality and poor prognosis in patients on hemodialysis. The purpose of this study was to investigate the prediction of overhydration and sarcopenia on mortality in patients on hemodialysis using a body composition monitor. We conducted a systematic review and meta-analysis using a random-effects model. We searched the Cochrane Central Register, OVID MEDLINE, EMBASE and PubMed databases for all studies published prior to December 9, 2016 and reviewed the reference lists of relevant reviews, registered trials and relevant conference proceedings. The overhydration group (fluid excess, >15% vs. the normohydration group) and the low lean tissue index group ( <10%) were compared with a reference group. Six trials, consisting of 29,469 patients, were included in the pooled analysis. The pooled hazard ratio for overall survival of the overhydration group, compared with the reference normohydration group was 1.798 (95% confidence interval [CI]: 1.53–2.804, *p* = .001). The hazard ratio for mortality in the low lean tissue index group was 1.533 (95% CI, 1.411–1.644; *p* = .001) in the random-effects model. The results from the most recent study showed the greatest heterogeneity in the sensitivity analysis. Low lean tissue index and overhydration, measured using a body composition monitor, were associated with a high mortality rate in patients on hemodialysis.

## Introduction

Maintaining adequate fluid volume and preventing sarcopenia are important goals when treating patients on hemodialysis (HD). Overhydration (OH) is defined as fluid excess greater than 15%, compared with normally hydrated people [1]. OH causes hypertension, left ventricular hypertrophy, pulmonary edema and heart failure; it is also associated with a high mortality and morbidity [2–4]. Alternatively, excessive fluid removal can increase the risk for intradialysis hypotension and contribute to a low dialysis efficiency [5]. In most dialysis centers, volume assessment is measured using changes in body weight, blood pressure (BP) and ultrafiltration rate. These tests are difficult to standardize and inaccurate.

Patients with a low lean tissue index (LTI) are in the bottom tenth percentile, compared with those in a reference population [6]. Patients with a low LTI have a significantly higher mortality than those with a normal LTI [1,7]. A low LTI is also associated with a low body mass index and low serum albumin [2]. Finally, a low LTI is an independent predictor of mortality and indicates malnutrition.

Recently, body composition monitors (BCMs) have been shown to be a noninvasive, fast, reproducible method for assessing body fluid status and composition in patients on HD [8,9]. BCMs determine the quantitative amount of OH and LTI by measuring tissue resistance and reactance at multiple frequencies [10,11]. However, it is not known whether these parameters, measured using a BCM predict of patient mortality.

We sought to conduct a systemic review and meta-analysis to evaluate the correlation of OH and low LTI values, measured using a BCM, on mortality in adults undergoing chronic HD. We compared patient mortality between patients on HD with OH and/or a low LTI, to that of patients on HD with normohydration and normal LTI.

## Materials and methods

This review was conducted in accordance with the Preferred Reporting Items for Systematic Reviews and Meta-Analyses (PRISMA) statement [12] (Supplementary Table 1 Checklist).

### Search strategy

Two researchers (SDH and JHL) independently performed comprehensive searches of the following databases for studies published from the database inception through December 9 2016: MEDLINE (via by PubMed), EMBASE, CINAHL, Web of Science and the Cochrane Central Register of Controlled Trials (CENTRAL) in the Cochrane Library. By using a highly sensitive search strategy to identify randomized controlled trials (RCTs) and observational cohort studies, we searched for: ‘kidney,’ ‘end stage renal disease,’ ‘dialysis,’ ‘hemodialysis,’ ‘peritoneal dialysis,’ ‘body composition monitor,’ ‘in body,’ ‘fluid overload,’ ‘lean tissue index,’ ‘overhydration,’ ‘mortality,’ and ‘electrode.’ We used combinations of these words. The search was limited to human studies, but not restricted to any particular language or publication date. Reference lists from all available review articles and RCTs were searched manually.

### Study selection

The review included RCTs of adults diagnosed with end-stage renal disease who were evaluated using a BCM and classified as OH and LTI (treatment groups) or normal hydration and LBI (control group). The review did not include any duplicate publications or sub-studies. We included all randomized and observational controlled trials published from January 1946 to December 2016. This included crossover, parallel arm and cluster trials. We also included all adult patients (age >18 years) who received chronic HD in an inpatient or outpatient setting, regardless of their BP levels at baseline. To avoid double counting patients included in more than one article by the same authors or research groups and patient recruitment periods were evaluated, if necessary the authors were contacted for clarification. Titles and abstracts of retrieved articles were independently evaluated by two researchers (SDH and JHL). Full-text articles were reviewed when the abstracts did not provide sufficient information regarding the inclusion and exclusion criteria. We independently evaluated the full text of each article and determined eligibility for inclusion in the review. Results were compared and discrepancies were resolved through discussion and if necessary, the views of a third researcher (JHS) was also included.

### Risk of bias assessment

The data from the included studies were extracted by one author (JHL) and reviewed by a second author (SDH). The publication year, country, type of study, number of participants, sex ratio, mean age, degree of hydration, LTI, BP, pulse wave velocity and relative fluid overload were collected from each study. The Newcastle-Ottawa Scale tawa Scale was used for cohort studies [13] and the Jadad scale was used to assess the risk of bias for each study [14].

### Calculate the total body water and the lean tissue index

The BCM - Body Composition Monitor expresses the body weight in terms of lean tissue mass (LTM- mainly muscle), adipose tissue mass (ATM-mainly fat) and overhydration (OH). The clinically relevant output parameters ‘Overhydration (OH)/Lean Tissue Mass (LTM)/Adipose Tissue Mass (ATM)’ are obtained by using two advanced validated physiological models: A volume model describing electrical conductance in a cell suspension enabling the total body water and extracellular water as well as the intracellular water (ICW) was to be calculated [9]. A body composition model calculating the three relevant body compartments overhydration, lean tissue and adipose tissue from ECW and TBW information [8]. Each of these compartments has a specific composition and contains a known quantity of water per mass of tissue. OH is almost 100% extracellular water, whereas the water of LTM and ATM consist of differing proportion of extracellular and intracellular water in addition to solid components. In a state of normal hydration with no excess fluid, lean tissue (mainly muscle) consists of 70% water whilst the remaining mass is protein and mineral. Adipose tissue (the majority of which is lipid) consists of 20% water. Excess fluid (overhydration) is almost 100% extracellular water.

### Primary outcomes

When using BCMs, OH was defined as fluid excess greater than 15%, compared with the normohydration group. LTI was defined as below the tenth percentile of a reference population. The primary outcome of interest was BCM use. Odds ratio (OR) for mortality, related to OH, was the primary outcome studied among those undergoing BCM measurements. LTI, BP and relative fluid overload were also analyzed as part of a subgroup analysis. Studies that used different standards—for instance an OH definition of 7%—or studies using a single regression analysis were excluded from the meta-analysis.

### Data extraction

Each of the reports that met the criteria for inclusion was read in detail by SDH, JHL and JHS; relevant details were summarized in standard extraction sheets. Results of data extraction were then compared and any discrepancy was resolved by discussion. When the same results were presented in more than one publication, we included the publication with the most complete results. If results were incomplete or unclear, we contacted the study authors for additional information [15]. We collected the following information from each trial: trial characteristics (author name, country, language, year of publication, number of countries, number of centers, inclusion and exclusion criteria), patient characteristics (number, patients completing follow-up, age, comorbidities and baseline BP), interventions and comparison characteristics (BCM, inbody and other measurements). We also collected information regarding funding sources, conflict of interest statements, consent and ethics approval.

### Data synthesis

The Comprehensive Meta-Analysis (CMA) software version 2.2.064 (Biostat Inc, Englewood, NJ) was used for this meta-analysis. We calculated the pooled complete resection rate and adverse event rate with 95% confidence intervals (CIs) from the enrolled studies. Heterogeneity was determined using the I^2^ test, which was developed by Higgins; this test measures the percentage of total variation across studies [16]. I^2^ was calculated with the following formula: I^2^ (%) = 100 × (Q-*df* )/Q, where Q is Cochrane’s heterogeneity statistic and df signifies the degree of freedom. Negative values for I^2^ were set to zero and an I^2^ value greater than 50% was considered substantially heterogeneous (range, 0–100%) [17]. Pooled-effect sizes with 95% CIs were calculated using a random effects model and the DerSimonian and Laird method [18]. These results were confirmed by the I^2^ test. A fixed effects model that included the inverse variance-weighted (Woolf’s) method was used in the sensitivity analyses, including cumulative and one-study-removed analyses, based on the assumption of a common effect size shared by the studies within each subgroup [19]. Significance was set at *p* = .05 in both models. Publication bias was evaluated with Begg’s funnel plot, Egger’s test of the intercept, Duval and Tweedie’s trim and fill, and Begg and Mazumdar’s rank correlation test [20–23].

### Quality of evidence assessment

We assessed the overall quality of the evidence for our primary outcome using an adapted Grading of Recommendations Assessment, Development and Evaluation (GRADE) approach [24]. The quality of the evidence for a specific outcome was based on performance versus the limitations of the study design, inconsistency of results, indirectness of evidence, imprecision of results and publication bias among all studies measuring that particular outcome. The overall quality of the evidence for the outcome was produced by combining assessments from all domains [25].

## Results

### Search results

**Figure 1.**
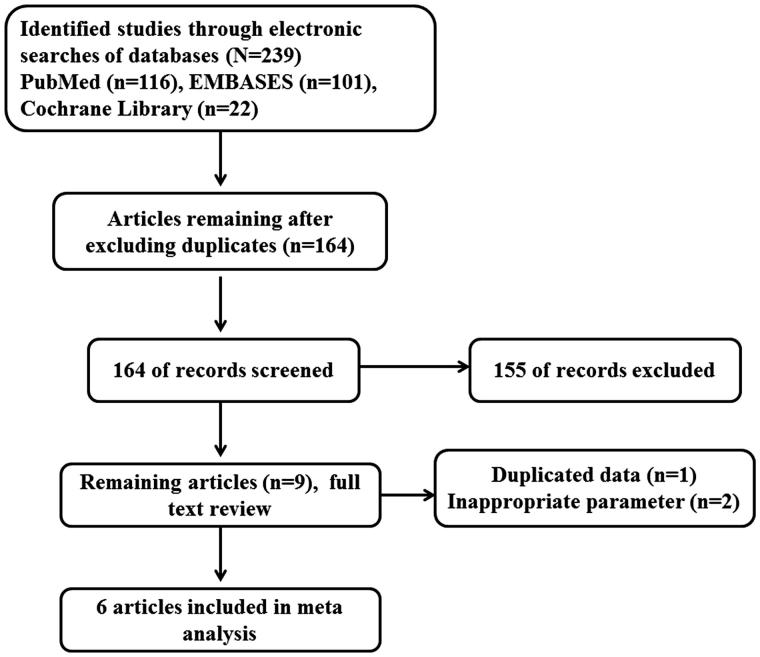
Flow diagram of the current systematic review.

Overall, 239 records were initially identified. Seventy-five records were excluded and the full texts of 164 studies were further evaluated for inclusion (Figure 1). We excluded 158 studies because they were published only in abstract form (*n* = 25), duplicates of other publications (*n* = 17), did not include proper data or subjects (*n* = 114) or provided more than one measurement (*n* = 2). After exclusions, one RCT and five observational studies including a total of 29,535 patients were included in our analysis.

### Study description

Six studies, including one RCT, were included (Table 1). The studies were conducted in various European countries, Portugal, Slovakia, the Republic of Korea, Germany and Turkey (one study from each location). The research period for these studies ranged from 2009 to 2015. Marcelli et al. included subjects from 15 countries, including Great Britain, and Wizemann et al. included subjects from three centers in Germany. In a longitudinal observational study, Caetano et al. analyzed the influence of OH, measured using a BCM, on mortality. Rosenberger et al. and Kim et al. published retrospective studies and Kim et al. used propensity scores. Onofriescu et al. conducted a RCT studying OH, but the sample size was small and conducted the sensitive analysis to identify whether the intervention toward the small sample got the same findings or not. The methodological quality of the included trials is shown in Table 2. There were five cohort studies and the mean number of stars for these trials was 7.8. The RCT that was included in the meta-analysis had two points.

**Table 1.** Characteristics of included studies.

			Normal BCM group (*n* = 14,263)				OH or low LTI (*n* = 15,272)							
References	Country	Type of study	Number of participants	Mean age (SD or range)	BMI	DM (%)	Number of participants	Mean age	BMI	DM	Follow-up Duration (month)	OR (95% CI)	Year	Analysis
Marcelli et al. [37]	European countries^a^	Cohort	12,776	62.2 ± 2.2	26.0	27.6	14608	63.3 ± 15.6	25.4	28.0	8.7 (38–12.4)	1.53 (1.40–1.66)	2015	Multinational cohort study
Caetano et al. [38]	Portugal	Cohort	697	65.4 ± 14.3	25.3	34.4	66	65.4 ± 14.3	22.9	47.0	12	2.21 (1.29–3.79)	2015	Multicenter longitudinal observational study
Rosenberger et al. [33]	Slovak Republic	Cohort	430	64.0 (54–75)	27.0	28.1	318	61.0 (53–69)	25.7	28.3	54	1.66 (1.10–2.49)	2014	Retrospective cohort study
Kim et al. [39]	Korea	Cohort	80	62.4 ± 11.2		61.3	160	65.6 ± 12.8		70.0	60	2.58 (1.15–5.79)	2015	Single-center retrospective study
Wizemann et al. [1]	Germany	Cohort	211	66.0 ± 15.2	25.8	32.0	58	65.0 ± 14.8	23.9	15.0	42	2.10 (1.28–3.44)	2009	Multicenter cohort study
Onofriescu et al. [40]	Turkey	RCT	69	54.0 ± 13	25.0	9.0	62	52.0 ± 13	24.3	10.0	30	0.9 (0.48–1.66)	2014	Randomized controlled parallel-group trial

BCM: body composition monitor; SD: standard deviation; RCT: randomized controlled trial; OH: overhydration; LTI: low lean tissue index; HR: hazard ratio.

a France, Hungary, Ireland, Italy, Poland, Portugal, Romania, Russia, Serbia, Slovakia, Slovenia, Spain, Sweden, Turkey, and the United Kingdom.

**Table 2.** Assessment of the risk of bias in each study using the Newcastle–Ottawa scale (NOS) and the Jadad scale^a^.

		Selection (0–4)				Comparability (0–2)			Outcome (0–3)			
Type of trials	Trials	REC	SNEC	AE	DO	SC		AF	AO	FU	AFU	Total
Cohort studies (NOS)	Marcelli et al.	★	★	★	★	★			★	★	★	8
	Caetano et al.	★	★	★	★	★			★	★	★	8
	Rosenberger et al.	★	★	★	★	★			★	★	★	8
	Kim et al.	★	★	★	★	★			★	★	★	8
	Wizemann et al.	★	★	★	★	★			★	★	★	7
		Randomization			Blinding							
		Mentioned	Appropriated		Mentioned		Appropriated		An account of all patients			
RCT (Jadad)	Onofriescu et al.	1	0		0		0		1			2

REC: representativeness of the exposed cohort; SNEC: selection of the non-exposed cohort; AE: ascertainment of exposure; DO: demonstration that the outcome of interest was not present at the start of the study; SC: study controls for age, sex and marital status; AF: study controls for any additional factors; AO: assessment of outcome; FU: was the follow-up sufficient for outcomes to occur; AFU: adequacy of the follow-up of cohorts; RCT: randomized controlled trial.

^a^A star means that the study satisfied the specified item. The absence of a star means that the specified item was not satisfied.

### Effect of interventions

**Figure 2.**
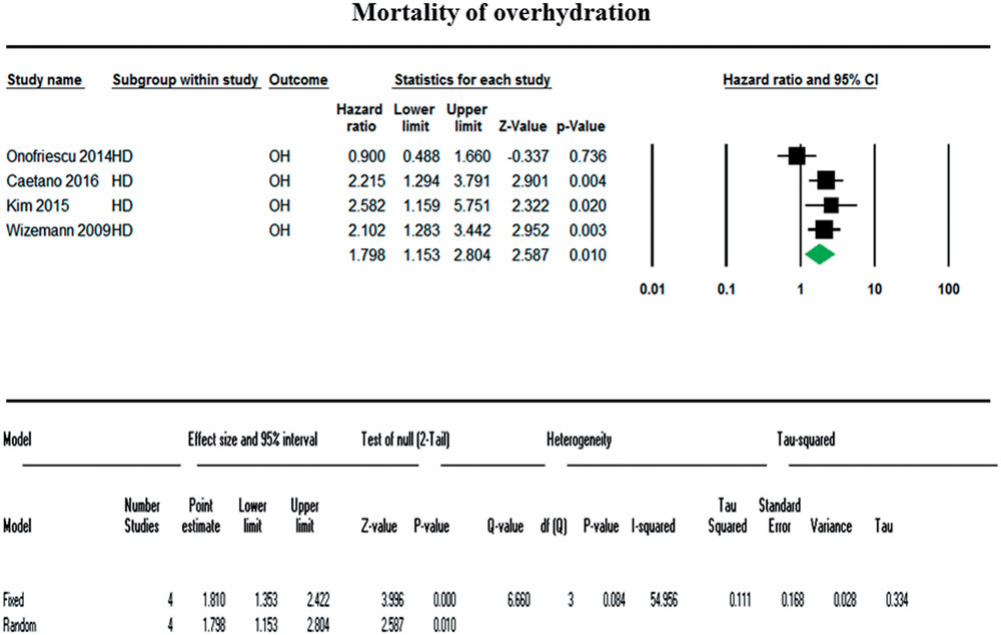
Forest plot comparing mortality between the body composition monitor use group and the control group for overhydration. A random-effects model pooling of the results showed that detection of OH using BCM was significantly associated with an increased mortality (OR, 1.79; 95% CI, 1.53–2.80).

**Figure 3.**
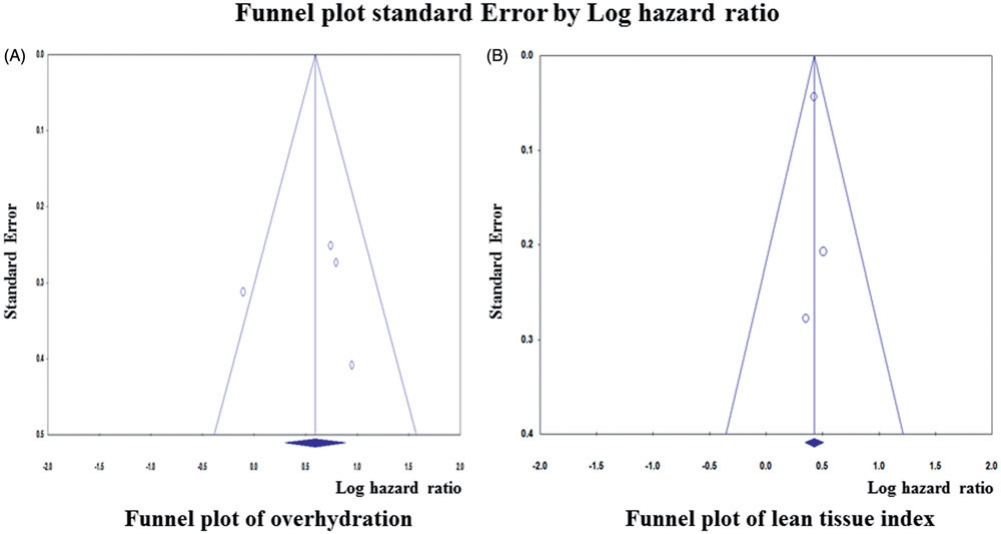
Funnel plots for overhydration and lean tissue index.

**Figure 4.**
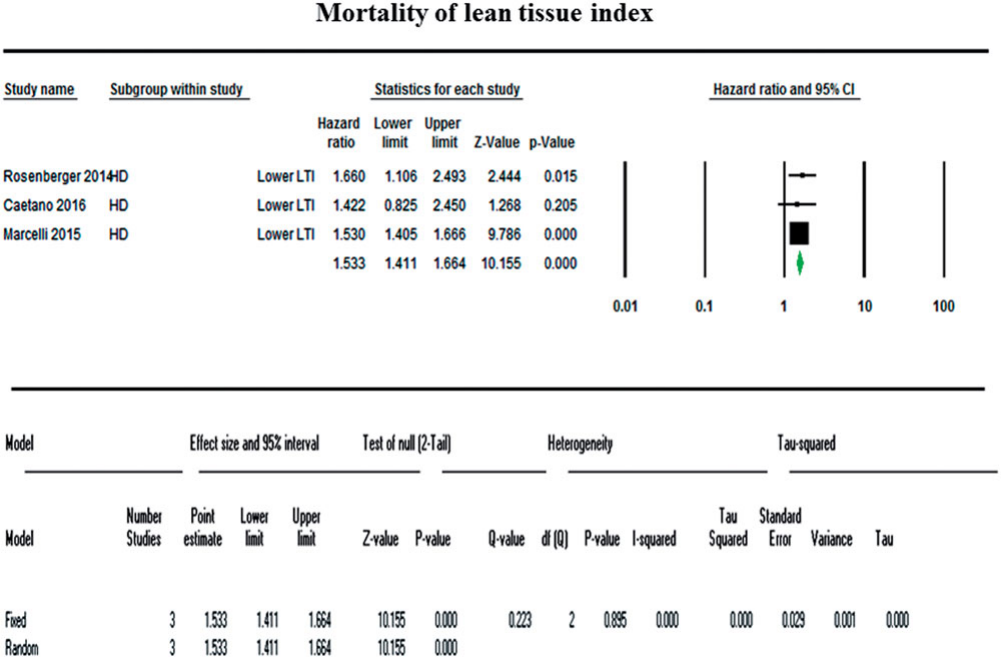
Forest plot comparing mortality between the body composition monitor use group and the control group for low lean tissue index. Low LTI measured using BCM was associated with a significantly increased mortality (OR, 1.533; 95% CI, 1.411–1.644).

A total of 15,272 patients were included in the OH and LTI groups measured using BCM and 14,263 patients in the normal (control) group. A random-effects model pooling of the results showed that detection of OH using BCM was significantly associated with an increased mortality (for all four studies, OR, 1.798; 95% CI, 1.53–2.804; Figure 2). Visual inspection of the forest plot and statistical tests demonstrated considerable heterogeneity among the studies (*p* = .084; I^2^ = 54.95%) (Figure 3(A)). The sensitivity analysis revealed that the result was robust and did not dependent on any individual study. The subgroup analysis including three studies of a fixed-effects model pooling of the results showed that low LTI measured using BCM was associated with a significantly increased mortality (OR, 1.533 for all three studies; 95% CI, 1.411–1.644; Figure 4). Forest plot and statistical tests also demonstrated considerable non-heterogeneity among the studies (*p* = .895; I^2^ = 0%) (Figure 3(B)). Possible asymmetry was found in the funnel plots. However, it was difficult to determine the publication bias of outcomes, due to the small number of the studies included. As a result of further evaluation, the BMI of the low LTI group was OR =1.008 (95% CI, 0.969–1.048) and the *p* values was 0.708. DM was also 1.054 (95% CI, 0.746–1.488) with a *p* value of.767. In the OH group, the OR ratio was 1.235 (95% CI, 0.678–2.248) and the *p* value was .490. However, Kim et al. reported cardiovascular events as a result. In contrast, cardiovascular event results of other studies were not reported.

## Discussion

This study met the methodological criteria proposed for systematic reviews and was conducted using a comprehensive search of relevant reviews, registered trials and relevant conference proceedings to avoid bias. The meta-analysis demonstrated that the OR for OH in patients on HD was 1.798 (CI: 1.153–2.804, *p* = .001) and that the HR for a low LTI in patients on HD was 1.533 (CI: 1.411–1.664, *p* = .001), compared with the control group.

To validate the above results, validation was confirmed from several studies. The BCM model has been validated in multicenter studies against the respective gold standards in healthy subjects and in hemodialysis patients. The comparison with gold standard methods showed excellent accordance (e.g. R2 (total body water) = 0.88; median 8 SD (total body water) = −0.17 + −2.7 liters] [26]. Lean Tissue Index (LTI) which is the lean tissue mass normalized to body height has also been validated against DEXA in 673 healthy subjects and patients (R^2^ = 0.78) [27].

Normal volume status contributes to a decreased mortality and morbidity in patients on HD. BCM measures body composition using a current of 5–1000 kHz to determine the electrical resistance of total body water and extracellular water. Its use is based on the principle that high frequency current passes through total body water, while low frequency current cannot penetrate cell membranes [28]. BCM can be used to measure parameters such as lean tissue mass, adipose tissue mass and OH noninvasively and quickly. Extensive validation of BCM testing has been performed in patients on HD [8,9].

Sustained fluid overload adversely affects the cardiovascular system by causing hypertension and left ventricular hypertrophy. Fluid overload in hemodialysis patients is associated with a higher mortality rate [1,3]. Even in patients with stage 4–5 chronic kidney disease, fluid overload has a higher predictive value for the need of dialysis and renal function decline than does diabetes mellitus [29]. Hur et al. reported an improvement in the left ventricular mass index and vascular stiffness of patients when fluid management was guided by BCM in a RCT. Fluid status optimization using BCM can maintain a normal fluid volume on a long-term basis, reducing the mortality rate and improving the quality-of-life of patients on HD [30,31].

The LTI is an important measurement related to the amount of skeletal muscle and reflects patients nutritional status. Malnutrition is defined as an imbalance between intake and utilization and results in altered metabolism, impaired function and a loss of body mass and skeletal muscle [32]. Nutritional status is an established risk factor for morbidity and mortality in the general population and patients on HD [33]. Skeletal muscle is considered the main source of protein in the body and protein is essential for antibody production, wound healing and white blood cell production during acute or chronic illnesses. If muscle is depleted, there is less protein to fuel these bodily functions, thereby enhancing the risk for disability and functional impairment while reducing muscle power and/or physical function [34]. Protein energy wasting is associated with an increased morbidity, mortality and an impaired quality-of-life. Several markers, including low body mass index (weight/height^2^), serum albumin, serum cholesterol and an elevated C-reactive protein, either as an isolated metric or incorporated as part of a score, have been previously associated with undernutrition in patients on HD [35]. However, these parameters have not been clearly related to prognosis in this patient population. LTI has recently been reported to be an easily measured parameter for lean mass that can be followed and monitored to assess risk and protein-energy wasting in patients on HD [36].

This meta-analysis has several strengths. The advantage of this review is the large sample size. The individual articles alone did not have enough statistical power because of the small number of patients included in each study. However, our meta-analysis has sufficient statistical power because 29,000 patients with the same condition were registered and analyzed. To our knowledge, this study is also the first meta-analysis of parameters obtained with BCM to evaluate body composition and fluid status in patients on HD. We found that patients with OH and low LTI, measured using BCM, have a higher mortality than those in the control group.

In the future, using BCM in dialysis centers may play a significant role in decreasing the mortality rate of patients on HD by maintaining normal volume and avoiding the development of a low LTI. Still, many patients on HD experience OH and/or a low LTI status. Dialysis clinicians need to strive to maintain normal hydration and reduce low LTI by avoiding nutrient deprivation. In addition, patients should be educated on balanced nutrition and dry weight maintenance. The medical staff should be involved in patient education and compliance monitoring with regard to these efforts. Currently, there are also an isotope dilution and other methods to measure hydration and LTI, but they are more difficult to perform clinically than BCM. We anticipate that new techniques that measure hydration status and LTI more accurately and easily than BCM will be developed in the future.

For the first time, this study includes high quality, multicenter studies with a large number of patients to assess the adverse effects of OH and low LTI, measured using BCM, in patients on HD.

This article has few limitations. The results are limited by the quality of the underlying studies included. Most of the included studies had a small number of subjects and short follow-up periods; long-term outcomes were not available among the included studies. While it was acceptable to use an RCT in the meta-analysis, the use of this cohort may represent a limitation. However, there have been few RCTs researching mortality, because BCM has only been recently developed. Therefore, we believe that this study is meaningful because it has a similar accuracy in predicting mortality than previous studies that have also used the cohort study method. Although the studies included in this study are large-scale studies, the sample sizes included in each study vary. Therefore, the importance of each research is not uniformly distributed. Among the studies included in our study, large sample sizes may show bias that has a greater impact on outcomes because of the large number of patients enrolled. This study did not include an analysis of the population of hemodialysis patients worldwide. Most studies included studies conducted in Europe and only one study included studies conducted in Asia.

In conclusion, low LTI and OH, measured using BCM, are effective indicators for predicting a high mortality rate in patients on HD.

## Supplementary Material

Supplementary Table
